# Crystal structure of 4-(4-meth­oxy­phen­yl)-4′,4′-dimethyl-3-*p*-tolyl-3′,4′-di­hydro-1′*H*,3*H*-spiro­[isoxazole-5,2′-naphthalen]-1′-one

**DOI:** 10.1107/S2056989015022033

**Published:** 2015-11-21

**Authors:** Mohamed Akhazzane, Ghali Al Houari, Mohamed El Yazidi, Mohamed Saadi, Lahcen El Ammari

**Affiliations:** a Laboratoire de Chimie Organique, Faculté des Sciences Dhar el Mahraz, Université Sidi Mohammed Ben Abdellah, Fès, Morocco; b Laboratoire de Chimie du Solide Appliquée, Faculté des Sciences, Université Mohammed V de Rabat, Avenue Ibn Battouta, BP 1014, Rabat, Morocco

**Keywords:** crystal structure, isoxazole, tetra­lone

## Abstract

In the title compound, C_28_H_27_NO_3_, the cyclo­hexa­none and isoxazole rings have envelope conformations, with the methyl­ene and spiro C atoms as the flaps, respectively. The mean plane of the isoxazole ring is inclined slightly to the *p*-tolyl ring, making a dihedral angle of 14.20 (9)°, and is nearly perpendicular to the mean plane through the tetra­lone moiety and to the meth­oxy­phenyl ring [dihedral angles = 83.41 (8) and 72.12 (9)°, respectively]. The crystal packing is stabilized mainly by van der Waals forces.

## Related literature   

For general background to 1,3-dipolar cyclo­addition reactions, see: Al Houari *et al.* (2008 ▸, 2010 ▸). For the structures of related compounds, see: Akhazzane *et al.* (2010 ▸, 2011 ▸); Mahfoud *et al.* (2015 ▸).
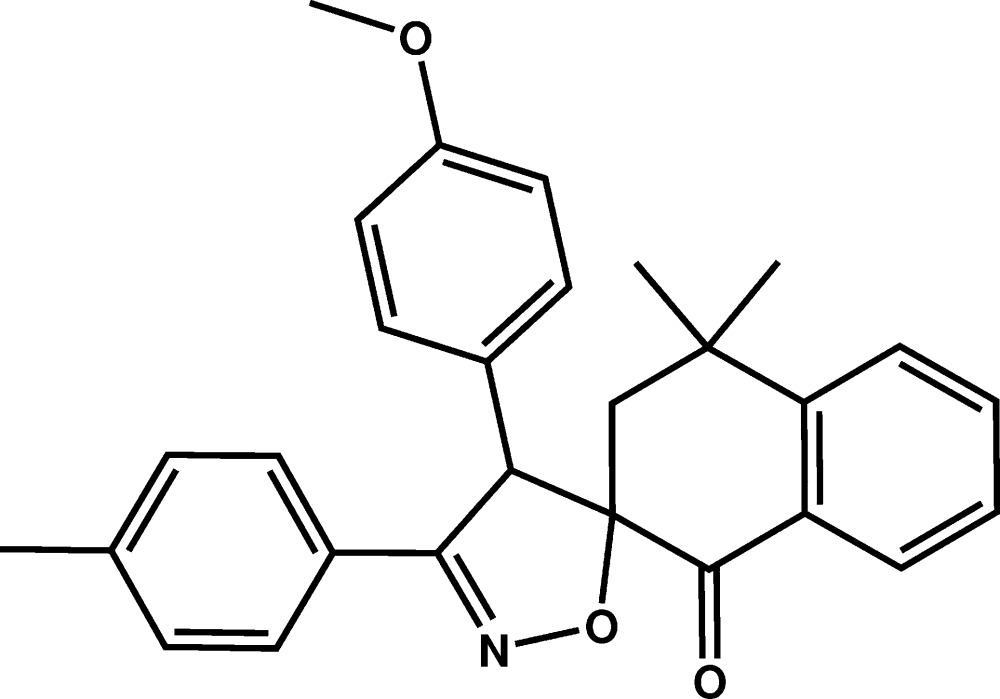


## Experimental   

### Crystal data  

C_28_H_27_NO_3_
*M* *_r_* = 425.50
Monoclinic, 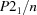

*a* = 10.2158 (8) Å
*b* = 12.9129 (10) Å
*c* = 17.6582 (14) Å
β = 103.801 (3)°
*V* = 2262.1 (3) Å^3^
*Z* = 4
Mo *K*α radiation
μ = 0.08 mm^−1^
*T* = 296 K
0.42 × 0.31 × 0.26 mm

### Data collection  

Bruker X8 APEX diffractometer
33809 measured reflections
5385 independent reflections
3283 reflections with *I* > 2σ(*I*)
*R*_int_ = 0.054

### Refinement  

*R*[*F* ^2^ > 2σ(*F* ^2^)] = 0.049
*wR*(*F* ^2^) = 0.129
*S* = 1.02
5385 reflections
289 parameters
H-atom parameters constrained
Δρ_max_ = 0.21 e Å^−3^
Δρ_min_ = −0.17 e Å^−3^

### 

Data collection: *APEX2* (Bruker, 2009 ▸); cell refinement: *SAINT* (Bruker, 2009 ▸); data reduction: *SAINT*; program(s) used to solve structure: *SHELXS97* (Sheldrick, 2008 ▸); program(s) used to refine structure: *SHELXL2014* (Sheldrick, 2015 ▸); molecular graphics: *ORTEP-3 for Windows* (Farrugia, 2012 ▸); software used to prepare material for publication: *PLATON* (Spek, 2009 ▸) and *publCIF* (Westrip, 2010 ▸).

## Supplementary Material

Crystal structure: contains datablock(s) I. DOI: 10.1107/S2056989015022033/rz5177sup1.cif

Structure factors: contains datablock(s) I. DOI: 10.1107/S2056989015022033/rz5177Isup2.hkl

Supporting information file. DOI: 10.1107/S2056989015022033/rz5177Isup3.cml

**Fig. 1.**
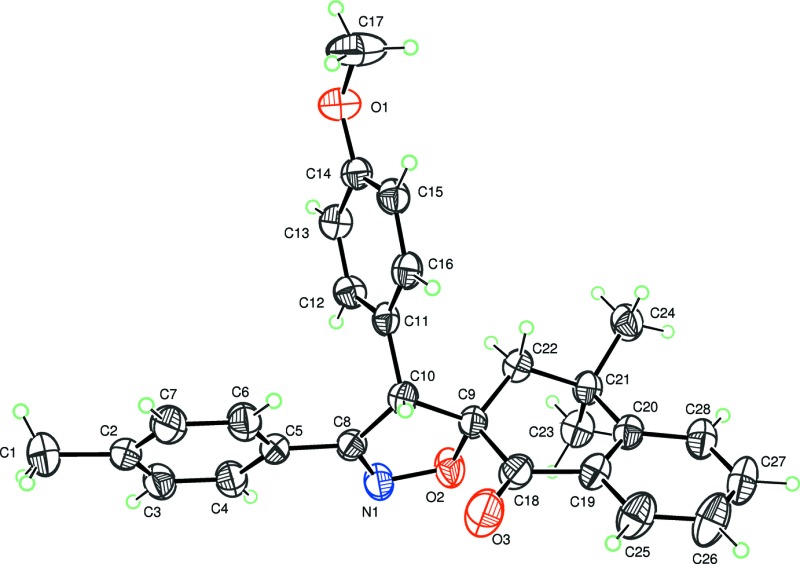
The molecular structure of the title compound with displacement ellipsoids drawn at the 50% probability level. H atoms are represented as small circles.

. DOI: 10.1107/S2056989015022033/rz5177fig1.tif
The mol­ecular structure of the title compound with displacement ellipsoids drawn at the 50% probability level. H atoms are represented as small circles.

CCDC reference: 1437668

Additional supporting information:  crystallographic information; 3D view; checkCIF report

## supplementary crystallographic information

### S1. Comment

In the context of our research concerning the approach of dipole-dipolarophile in 1,3-dipolar cycloaddition, we have already studied the case where the dipole is an arylnitriloxide and the dipolarophiles are 2-arylidenes of tetralone (systematic name: 3,4-dihydronaphthalen-1-one) substituted by an anisopropyl group in position 4 (Al Houari *et al.*, 2008; Al Houari *et al.*, 2010). We have shown that the ring closure reaction is highly regiselective and also highly diastereoselective. The relative configuration and conformation of the products have been determined by means of proton magnetic resonance measurements. In this paper we describe the regiochemistry in the reaction of *para*-tolylnitriloxide with (*E*)-2-(4-methoxybenzylidene)-4,4-dimethyl-3,4-dihydronaphthalen-1(2*H*)-one, as a continuation of the investigation on dihydronaphthalene derivatives (Akhazzane *et al.*, 2010, Akhazzane *et al.*, 2011, Mahfoud *et al.*, 2015).

The molecule of the title compound is build up from a tetralone moiety linked to an isoxazole ring which is connected to a *p*-tolyl ring and to a methoxyphenyl group in axial position as shown in Fig. 1. The cyclohexanone and the isoxazole rings adopt an envelope conformation with atoms C22 and C9 (spiro C atom) as the flap, respectively. The puckering parameters (Cremer & Pople, 1975) are: Q_T_ = 0.413 (2) Å, θ = 122.0 (3)° and φ = 111.1 (3)° for the cyclohexanone ring; q2 = 0.0931 (17) Å, φ2 = 329.4 (11)° for the isoxazole ring. The mean planes through the tetralone moiety, the methoxyphenyl ring and the *p*-tolyl ring are inclined to the mean plane of the isoxazole ring by dihedral angles of 83.41 (8), 72.12 (9) and 14.20 (9)°, respectively. In the crystal, packing is enforced only by van der Waals interactions.

### S2. Experimental

In a 100 ml flask, 2 mmol of the arylidene 2-(4-methoxybenzylidene)-4,4-dimethyl-3,4-dihydronaphthalen-1(2*H*)-one and 2.4 mmol of *p*-tolyloxime were dissolved in 20 ml chloroform. The mixture was cooled to 273 K under magnetic stirring in an ice bath. Then 15 ml of bleach at 18° (chlorometric degree) was added in small doses without exceeding 278 K. The mixture was left under magnetic stirring for 16 h at room temperature, then washed with water until the pH was neutral and dried on sodium sulfate. The solvent was evaporated with a rotating evaporator and the oily residue was dissolved in ethanol. The resulting residue was recrystallized from ethanol to afford the title compound as colourless needles crystals on slow evaporation of the solvent (yield: 58%; m. p: 443 K).

### S3. Refinement

One reflection (0 1 1) affected by the beamstop was removed in the cycles of refinement. All H atoms were located in a difference Fourier map and treated as riding, with C–H = 0.96–0.98 Å and with *U*_iso_(H) = 1.2 *U*_eq_(C) or 1.5 *U*_eq_(C) for methyl H atoms.

### Crystal data

**Table:**

C_28_H_27_NO_3_	*D*_x_ = 1.249 Mg m^−^^3^
*M**_r_* = 425.50	Melting point: 443 K
Monoclinic, *P*2_1_/*n*	Mo *K*α radiation, λ = 0.71073 Å
*a* = 10.2158 (8) Å	Cell parameters from 5385 reflections
*b* = 12.9129 (10) Å	θ = 2.4–27.9°
*c* = 17.6582 (14) Å	µ = 0.08 mm^−^^1^
β = 103.801 (3)°	*T* = 296 K
*V* = 2262.1 (3) Å^3^	Block, colourless
*Z* = 4	0.42 × 0.31 × 0.26 mm
*F*(000) = 904	

### Data collection

**Table:**

Bruker X8 APEX diffractometer	3283 reflections with *I* > 2σ(*I*)
Radiation source: fine-focus sealed tube	*R*_int_ = 0.054
Graphite monochromator	θ_max_ = 27.9°, θ_min_ = 2.4°
φ and ω scans	*h* = −13→12
33809 measured reflections	*k* = −16→16
5385 independent reflections	*l* = −23→23

### Refinement

**Table:**

Refinement on *F*^2^	0 restraints
Least-squares matrix: full	Hydrogen site location: inferred from neighbouring sites
*R*[*F*^2^ > 2σ(*F*^2^)] = 0.049	H-atom parameters constrained
*wR*(*F*^2^) = 0.129	*w* = 1/[σ^2^(*F*_o_^2^) + (0.050*P*)^2^ + 0.5224*P*] where *P* = (*F*_o_^2^ + 2*F*_c_^2^)/3
*S* = 1.02	(Δ/σ)_max_ < 0.001
5385 reflections	Δρ_max_ = 0.21 e Å^−^^3^
289 parameters	Δρ_min_ = −0.17 e Å^−^^3^

### Special details

**Table:**

Geometry. All e.s.d.'s (except the e.s.d. in the dihedral angle between two l.s. planes) are estimated using the full covariance matrix. The cell e.s.d.'s are taken into account individually in the estimation of e.s.d.'s in distances, angles and torsion angles; correlations between e.s.d.'s in cell parameters are only used when they are defined by crystal symmetry. An approximate (isotropic) treatment of cell e.s.d.'s is used for estimating e.s.d.'s involving l.s. planes.

### Fractional atomic coordinates and isotropic or equivalent isotropic displacement parameters (Å^2^)

**Table:**

	*x*	*y*	*z*	*U*_iso_*/*U*_eq_	
C1	0.9515 (2)	0.7328 (2)	0.36467 (15)	0.0826 (8)	
H1A	0.9351	0.6936	0.3171	0.124*	
H1B	0.9704	0.8035	0.3542	0.124*	
H1C	1.0273	0.7039	0.4015	0.124*	
C2	0.8284 (2)	0.72855 (16)	0.39799 (12)	0.0533 (5)	
C3	0.7148 (2)	0.67399 (16)	0.36081 (12)	0.0538 (5)	
H3	0.7133	0.6416	0.3136	0.065*	
C4	0.6036 (2)	0.66646 (14)	0.39201 (11)	0.0472 (5)	
H4	0.5289	0.6287	0.3659	0.057*	
C5	0.60251 (17)	0.71487 (13)	0.46221 (10)	0.0384 (4)	
C6	0.71442 (19)	0.77192 (16)	0.49867 (12)	0.0523 (5)	
H6	0.7152	0.8060	0.5452	0.063*	
C7	0.8254 (2)	0.77869 (18)	0.46652 (13)	0.0599 (6)	
H7	0.8994	0.8179	0.4917	0.072*	
C8	0.48550 (17)	0.70665 (13)	0.49692 (10)	0.0368 (4)	
C9	0.33554 (17)	0.71799 (13)	0.57913 (10)	0.0369 (4)	
C10	0.48673 (16)	0.73339 (12)	0.58022 (10)	0.0356 (4)	
H10	0.5078	0.8072	0.5880	0.043*	
C11	0.58808 (16)	0.67307 (12)	0.64087 (9)	0.0338 (4)	
C12	0.62936 (17)	0.57313 (13)	0.62684 (10)	0.0394 (4)	
H12	0.5945	0.5421	0.5787	0.047*	
C13	0.72088 (17)	0.52002 (13)	0.68328 (10)	0.0407 (4)	
H13	0.7477	0.4538	0.6728	0.049*	
C14	0.77340 (17)	0.56424 (13)	0.75562 (10)	0.0374 (4)	
C15	0.73391 (17)	0.66315 (13)	0.77078 (10)	0.0399 (4)	
H15	0.7682	0.6937	0.8191	0.048*	
C16	0.64284 (17)	0.71604 (13)	0.71310 (10)	0.0382 (4)	
H16	0.6177	0.7828	0.7234	0.046*	
C17	0.9263 (3)	0.55048 (19)	0.88033 (13)	0.0815 (8)	
H17A	0.9871	0.5016	0.9113	0.122*	
H17B	0.9755	0.6110	0.8718	0.122*	
H17C	0.8585	0.5697	0.9072	0.122*	
C18	0.26491 (19)	0.82319 (14)	0.57550 (11)	0.0463 (5)	
C19	0.15232 (17)	0.83574 (13)	0.61336 (10)	0.0409 (4)	
C20	0.09907 (17)	0.75196 (14)	0.64651 (10)	0.0392 (4)	
C21	0.15675 (17)	0.64320 (13)	0.64679 (10)	0.0388 (4)	
C22	0.30500 (17)	0.64932 (13)	0.64192 (10)	0.0393 (4)	
H22A	0.3582	0.6733	0.6919	0.047*	
H22B	0.3352	0.5798	0.6341	0.047*	
C23	0.07011 (19)	0.58162 (15)	0.57859 (12)	0.0509 (5)	
H23A	0.1059	0.5129	0.5784	0.076*	
H23B	−0.0208	0.5779	0.5844	0.076*	
H23C	0.0711	0.6155	0.5304	0.076*	
C24	0.1549 (2)	0.58621 (16)	0.72334 (12)	0.0543 (5)	
H24A	0.1916	0.5180	0.7222	0.081*	
H24B	0.2081	0.6240	0.7667	0.081*	
H24C	0.0638	0.5812	0.7287	0.081*	
C25	0.0964 (2)	0.93444 (15)	0.61386 (14)	0.0593 (6)	
H25	0.1330	0.9899	0.5924	0.071*	
C26	−0.0122 (2)	0.95033 (17)	0.64576 (15)	0.0700 (7)	
H26	−0.0484	1.0163	0.6465	0.084*	
C27	−0.0667 (2)	0.86782 (18)	0.67658 (14)	0.0627 (6)	
H27	−0.1411	0.8780	0.6976	0.075*	
C28	−0.01280 (19)	0.77072 (16)	0.67676 (12)	0.0519 (5)	
H28	−0.0518	0.7159	0.6976	0.062*	
N1	0.37202 (15)	0.67459 (12)	0.45609 (9)	0.0468 (4)	
O1	0.86366 (13)	0.50485 (9)	0.80727 (7)	0.0510 (3)	
O2	0.27756 (12)	0.67119 (11)	0.50248 (7)	0.0525 (4)	
O3	0.30170 (16)	0.89402 (12)	0.53998 (11)	0.0820 (6)	

### Atomic displacement parameters (Å^2^)

**Table:**

	*U*^11^	*U*^22^	*U*^33^	*U*^12^	*U*^13^	*U*^23^
C1	0.0597 (15)	0.117 (2)	0.0819 (18)	0.0152 (14)	0.0383 (14)	0.0347 (16)
C2	0.0439 (12)	0.0670 (13)	0.0533 (13)	0.0142 (10)	0.0199 (10)	0.0249 (11)
C3	0.0618 (14)	0.0563 (12)	0.0498 (12)	0.0125 (10)	0.0264 (11)	0.0073 (10)
C4	0.0490 (12)	0.0469 (10)	0.0496 (11)	0.0009 (9)	0.0193 (9)	0.0017 (9)
C5	0.0362 (10)	0.0411 (9)	0.0392 (10)	0.0050 (8)	0.0113 (8)	0.0065 (8)
C6	0.0426 (11)	0.0713 (14)	0.0437 (11)	−0.0038 (10)	0.0115 (9)	0.0002 (10)
C7	0.0393 (12)	0.0838 (15)	0.0555 (13)	−0.0092 (11)	0.0088 (10)	0.0129 (12)
C8	0.0352 (10)	0.0366 (9)	0.0390 (9)	0.0017 (7)	0.0096 (8)	0.0007 (7)
C9	0.0335 (9)	0.0402 (9)	0.0380 (10)	0.0011 (7)	0.0105 (8)	−0.0035 (7)
C10	0.0333 (9)	0.0347 (8)	0.0396 (10)	−0.0019 (7)	0.0102 (8)	−0.0026 (7)
C11	0.0288 (9)	0.0377 (9)	0.0372 (9)	−0.0040 (7)	0.0122 (7)	−0.0031 (7)
C12	0.0425 (10)	0.0396 (9)	0.0354 (9)	−0.0043 (8)	0.0077 (8)	−0.0077 (7)
C13	0.0443 (11)	0.0323 (8)	0.0460 (11)	−0.0020 (7)	0.0118 (9)	−0.0048 (8)
C14	0.0331 (9)	0.0384 (9)	0.0412 (10)	−0.0053 (7)	0.0096 (8)	0.0031 (8)
C15	0.0381 (10)	0.0460 (10)	0.0358 (9)	−0.0060 (8)	0.0089 (8)	−0.0078 (8)
C16	0.0366 (10)	0.0363 (9)	0.0436 (10)	−0.0007 (7)	0.0137 (8)	−0.0072 (8)
C17	0.101 (2)	0.0679 (15)	0.0544 (14)	0.0168 (14)	−0.0229 (13)	−0.0062 (12)
C18	0.0386 (10)	0.0429 (10)	0.0585 (12)	0.0028 (8)	0.0136 (9)	0.0073 (9)
C19	0.0330 (9)	0.0402 (9)	0.0492 (11)	0.0005 (7)	0.0091 (8)	−0.0073 (8)
C20	0.0326 (9)	0.0446 (9)	0.0399 (10)	−0.0021 (8)	0.0075 (8)	−0.0086 (8)
C21	0.0343 (9)	0.0397 (9)	0.0444 (10)	−0.0030 (7)	0.0129 (8)	−0.0030 (8)
C22	0.0355 (10)	0.0382 (9)	0.0451 (10)	0.0016 (7)	0.0110 (8)	−0.0013 (8)
C23	0.0451 (11)	0.0459 (11)	0.0618 (13)	−0.0068 (9)	0.0129 (10)	−0.0090 (9)
C24	0.0533 (13)	0.0570 (12)	0.0576 (13)	−0.0018 (10)	0.0234 (10)	0.0064 (10)
C25	0.0500 (12)	0.0404 (10)	0.0895 (17)	0.0010 (9)	0.0206 (12)	−0.0065 (10)
C26	0.0581 (14)	0.0495 (12)	0.107 (2)	0.0096 (11)	0.0299 (14)	−0.0218 (13)
C27	0.0471 (13)	0.0689 (14)	0.0782 (16)	0.0077 (11)	0.0271 (12)	−0.0192 (12)
C28	0.0424 (11)	0.0601 (12)	0.0575 (13)	0.0005 (9)	0.0206 (10)	−0.0064 (10)
N1	0.0397 (9)	0.0613 (10)	0.0417 (9)	−0.0045 (7)	0.0145 (7)	−0.0069 (8)
O1	0.0552 (8)	0.0442 (7)	0.0473 (8)	0.0008 (6)	−0.0006 (6)	0.0016 (6)
O2	0.0368 (7)	0.0785 (9)	0.0437 (7)	−0.0124 (7)	0.0127 (6)	−0.0136 (7)
O3	0.0735 (11)	0.0620 (9)	0.1266 (15)	0.0204 (8)	0.0559 (11)	0.0408 (10)

### Geometric parameters (Å, º)

**Table:**

C1—C2	1.511 (3)	C15—H15	0.9300
C1—H1A	0.9600	C16—H16	0.9300
C1—H1B	0.9600	C17—O1	1.424 (2)
C1—H1C	0.9600	C17—H17A	0.9600
C2—C7	1.379 (3)	C17—H17B	0.9600
C2—C3	1.382 (3)	C17—H17C	0.9600
C3—C4	1.379 (3)	C18—O3	1.218 (2)
C3—H3	0.9300	C18—C19	1.471 (3)
C4—C5	1.391 (2)	C19—C25	1.398 (3)
C4—H4	0.9300	C19—C20	1.400 (2)
C5—C6	1.383 (3)	C20—C28	1.393 (2)
C5—C8	1.471 (2)	C20—C21	1.523 (2)
C6—C7	1.386 (3)	C21—C23	1.535 (2)
C6—H6	0.9300	C21—C22	1.539 (2)
C7—H7	0.9300	C21—C24	1.543 (3)
C8—N1	1.279 (2)	C22—H22A	0.9700
C8—C10	1.508 (2)	C22—H22B	0.9700
C9—O2	1.472 (2)	C23—H23A	0.9600
C9—C22	1.509 (2)	C23—H23B	0.9600
C9—C18	1.532 (2)	C23—H23C	0.9600
C9—C10	1.553 (2)	C24—H24A	0.9600
C10—C11	1.516 (2)	C24—H24B	0.9600
C10—H10	0.9800	C24—H24C	0.9600
C11—C16	1.381 (2)	C25—C26	1.374 (3)
C11—C12	1.398 (2)	C25—H25	0.9300
C12—C13	1.376 (2)	C26—C27	1.373 (3)
C12—H12	0.9300	C26—H26	0.9300
C13—C14	1.385 (2)	C27—C28	1.369 (3)
C13—H13	0.9300	C27—H27	0.9300
C14—O1	1.367 (2)	C28—H28	0.9300
C14—C15	1.385 (2)	N1—O2	1.4077 (18)
C15—C16	1.385 (2)		
			
C2—C1—H1A	109.5	C15—C16—H16	118.9
C2—C1—H1B	109.5	O1—C17—H17A	109.5
H1A—C1—H1B	109.5	O1—C17—H17B	109.5
C2—C1—H1C	109.5	H17A—C17—H17B	109.5
H1A—C1—H1C	109.5	O1—C17—H17C	109.5
H1B—C1—H1C	109.5	H17A—C17—H17C	109.5
C7—C2—C3	117.65 (18)	H17B—C17—H17C	109.5
C7—C2—C1	121.3 (2)	O3—C18—C19	121.48 (17)
C3—C2—C1	121.1 (2)	O3—C18—C9	119.15 (17)
C4—C3—C2	121.63 (19)	C19—C18—C9	119.35 (15)
C4—C3—H3	119.2	C25—C19—C20	120.11 (17)
C2—C3—H3	119.2	C25—C19—C18	117.94 (17)
C3—C4—C5	120.47 (19)	C20—C19—C18	121.93 (16)
C3—C4—H4	119.8	C28—C20—C19	117.51 (17)
C5—C4—H4	119.8	C28—C20—C21	120.75 (16)
C6—C5—C4	118.20 (17)	C19—C20—C21	121.68 (15)
C6—C5—C8	120.53 (16)	C20—C21—C23	108.96 (15)
C4—C5—C8	121.27 (16)	C20—C21—C22	109.74 (14)
C5—C6—C7	120.58 (19)	C23—C21—C22	111.93 (14)
C5—C6—H6	119.7	C20—C21—C24	110.92 (15)
C7—C6—H6	119.7	C23—C21—C24	108.35 (15)
C2—C7—C6	121.4 (2)	C22—C21—C24	106.94 (15)
C2—C7—H7	119.3	C9—C22—C21	116.73 (15)
C6—C7—H7	119.3	C9—C22—H22A	108.1
N1—C8—C5	120.37 (16)	C21—C22—H22A	108.1
N1—C8—C10	114.79 (15)	C9—C22—H22B	108.1
C5—C8—C10	124.83 (15)	C21—C22—H22B	108.1
O2—C9—C22	109.03 (13)	H22A—C22—H22B	107.3
O2—C9—C18	104.09 (14)	C21—C23—H23A	109.5
C22—C9—C18	111.95 (14)	C21—C23—H23B	109.5
O2—C9—C10	104.17 (13)	H23A—C23—H23B	109.5
C22—C9—C10	116.45 (14)	C21—C23—H23C	109.5
C18—C9—C10	110.12 (14)	H23A—C23—H23C	109.5
C8—C10—C11	114.65 (14)	H23B—C23—H23C	109.5
C8—C10—C9	100.47 (13)	C21—C24—H24A	109.5
C11—C10—C9	116.92 (14)	C21—C24—H24B	109.5
C8—C10—H10	108.1	H24A—C24—H24B	109.5
C11—C10—H10	108.1	C21—C24—H24C	109.5
C9—C10—H10	108.1	H24A—C24—H24C	109.5
C16—C11—C12	117.49 (15)	H24B—C24—H24C	109.5
C16—C11—C10	120.28 (15)	C26—C25—C19	120.7 (2)
C12—C11—C10	122.23 (15)	C26—C25—H25	119.7
C13—C12—C11	120.88 (16)	C19—C25—H25	119.7
C13—C12—H12	119.6	C27—C26—C25	119.34 (19)
C11—C12—H12	119.6	C27—C26—H26	120.3
C12—C13—C14	120.61 (16)	C25—C26—H26	120.3
C12—C13—H13	119.7	C28—C27—C26	120.7 (2)
C14—C13—H13	119.7	C28—C27—H27	119.7
O1—C14—C15	124.73 (16)	C26—C27—H27	119.7
O1—C14—C13	115.80 (15)	C27—C28—C20	121.7 (2)
C15—C14—C13	119.47 (16)	C27—C28—H28	119.2
C16—C15—C14	119.23 (16)	C20—C28—H28	119.2
C16—C15—H15	120.4	C8—N1—O2	109.70 (14)
C14—C15—H15	120.4	C14—O1—C17	117.26 (15)
C11—C16—C15	122.30 (16)	N1—O2—C9	109.94 (12)
C11—C16—H16	118.9		
