# Supplementary figures and images for: Crystal structure of 4-(4-meth­oxy­phen­yl)-4′,4′-dimethyl-3-p-tolyl-3′,4′-di­hydro-1′H,3H-spiro­[isoxazole-5,2′-naphthalen]-1′-one
Source: Acta Crystallogr E Crystallogr Commun. 2015 Nov 21;71(Pt 12):o981. doi: 10.1107/S2056989015022033 (PMC4719934; doi:10.1107/S2056989015022033)

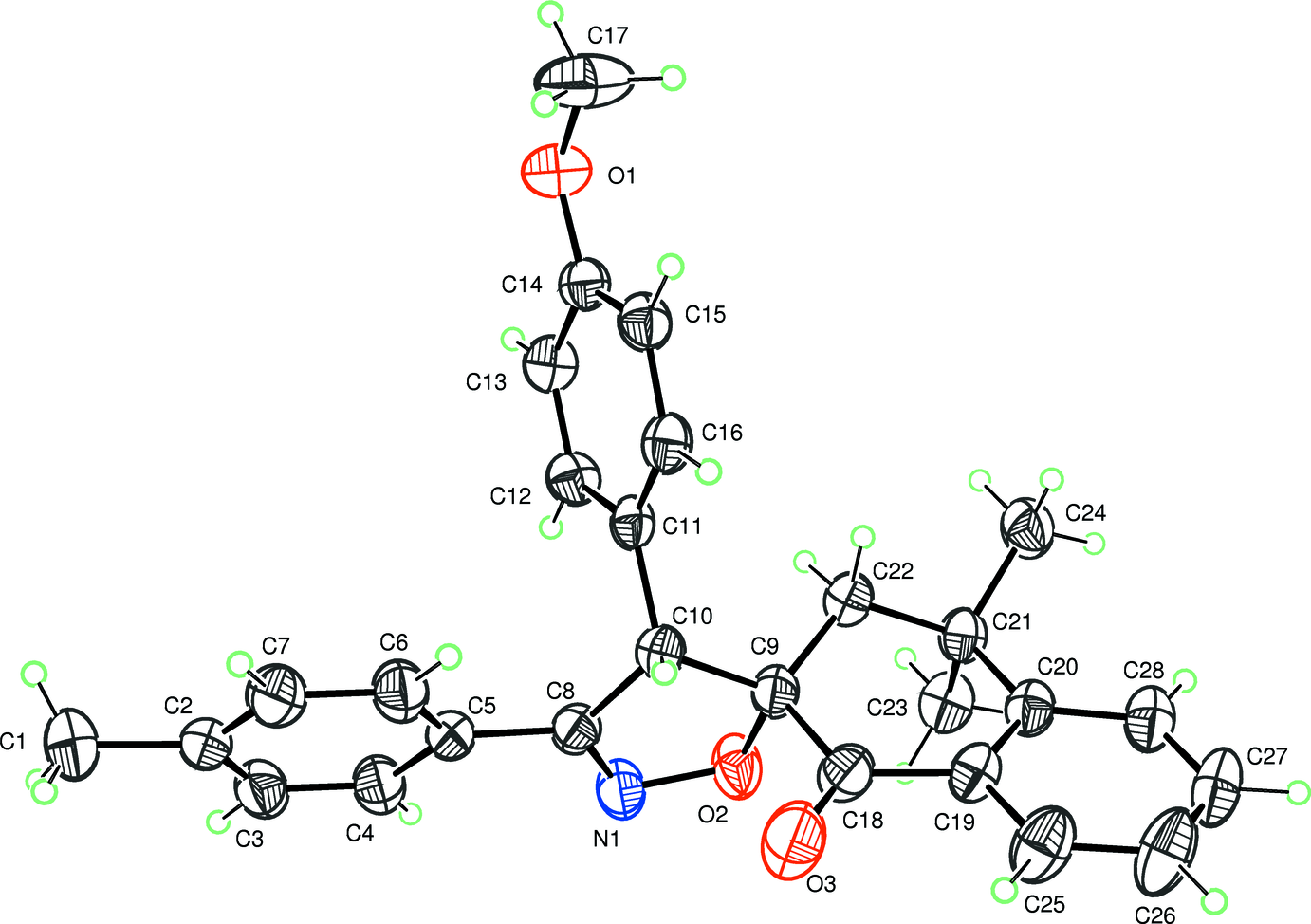

Supplement: Supplementary file 4 [file e-71-0o981-fig1.tif]
